# Metabolic pathways associated with right ventricular adaptation to pulmonary hypertension: 3D analysis of cardiac magnetic resonance imaging

**DOI:** 10.1093/ehjci/jey175

**Published:** 2018-12-07

**Authors:** Mark I Attard, Timothy J W Dawes, Antonio de Marvao, Carlo Biffi, Wenzhe Shi, John Wharton, Christopher J Rhodes, Pavandeep Ghataorhe, J Simon R Gibbs, Luke S G E Howard, Daniel Rueckert, Martin R Wilkins, Declan P O’Regan

**Affiliations:** 1 MRC London Institute of Medical Sciences, Du Cane Road, London, UK; 2 Division of Experimental Medicine, Department of Medicine, Imperial College London, Du Cane Road, London, UK; 3 Royal Brompton Cardiovascular Research Centre, National Heart & Lung Institute, Imperial College London, Dovehouse Street, London, UK; 4 Department of Computing, Imperial College London, South Kensington Campus, Queen’s Gate, London, UK; 5 Imperial College Healthcare NHS Trust, Du Cane Road, London, UK

**Keywords:** pulmonary hypertension, metabolomics, wall stress, cardiac magnetic resonance imaging, image segmentation, machine learning

## Abstract

**Aims:**

We sought to identify metabolic pathways associated with right ventricular (RV) adaptation to pulmonary hypertension (PH). We evaluated candidate metabolites, previously associated with survival in pulmonary arterial hypertension, and used automated image segmentation and parametric mapping to model their relationship to adverse patterns of remodelling and wall stress.

**Methods and results:**

In 312 PH subjects (47.1% female, mean age 60.8 ± 15.9 years), of which 182 (50.5% female, mean age 58.6 ± 16.8 years) had metabolomics, we modelled the relationship between the RV phenotype, haemodynamic state, and metabolite levels. Atlas-based segmentation and co-registration of cardiac magnetic resonance imaging was used to create a quantitative 3D model of RV geometry and function—including maps of regional wall stress. Increasing mean pulmonary artery pressure was associated with hypertrophy of the basal free wall (*β* = 0.29) and reduced relative wall thickness (*β* = −0.38), indicative of eccentric remodelling. Wall stress was an independent predictor of all-cause mortality (hazard ratio = 1.27, *P *=* *0.04). Six metabolites were significantly associated with elevated wall stress (*β* = 0.28–0.34) including increased levels of tRNA-specific modified nucleosides and fatty acid acylcarnitines, and decreased levels (*β* = −0.40) of sulfated androgen.

**Conclusion:**

Using computational image phenotyping, we identify metabolic profiles, reporting on energy metabolism and cellular stress-response, which are associated with adaptive RV mechanisms to PH.

## Introduction

Right ventricular (RV) failure complicates a diverse group of cardiorespiratory diseases. It is the strongest determinant of survival in pulmonary arterial hypertension (PAH), and independently predicts outcome in pulmonary hypertension (PH) secondary to left heart failure, chronic lung disease, or thrombo-embolic disease.^1^ Initially, there is a compensatory phase where the RV maintains efficient energy transfer by an adaptive increase in wall thickness (WT) and contractility,^2^ until the more advanced stages of disease when the RV dilates, wall stress rises, and ventriculo-arterial uncoupling occurs leading to heart failure.^3^ However, RV failure associated with PH is not simply a predictable consequence of increased afterload. Patients may show a progressive decline in RV function despite a therapeutic response in pulmonary vascular resistance,^4^ and data from animal models suggest a role for circulating mediators that interfere with adaptive RV mechanisms to elevated mechanical stress.^5^

It has recently been recognized that translational regulation and energy metabolism are disturbed in PAH and that monitoring of plasma metabolites that report on these pathways could be useful to assess disease progression and response to therapy.^6^ While metabolomics provides novel molecular insights into the pathogenesis of disease it is unknown if metabolic phenotyping, as well as predicting survival, could identify key pathways for mechanisms that influence myocardial adaptation to afterload. To examine this, we developed an automated method for 3D biomechanical modelling of the right ventricle using clinical cardiac magnetic resonance imaging (CMR)—and combined this with robust statistical techniques for mapping the strength of association between circulating biomarkers and regional wall stress. Our aim was to determine if molecular profiles, known to be predictive of survival, are associated with evidence of adverse RV remodelling and biomechanical stress in patients with PH.

## Methods

### Study population

The study was approved by the Heath Research Authority and all participants gave written informed consent. Patients referred between 2002 and 2015 to Imperial College Healthcare NHS Trust (London, UK), Hammersmith Hospital for routine diagnostic assessment of PH including cardiac imaging were included. A diagnosis of PH was made according to standard guidelines with a resting mean pulmonary artery pressure (mPAP) ≥25 mmHg by right heart catheterization (RHC),^7^ performed using standard Swan Ganz catheters introduced via an internal jugular, subclavian, or femoral vein approach. Exclusion criteria were PH due to congenital heart disease, previous pulmonary endarterectomy, or more than 6 months between baseline investigations. Disease classification was according to the Dana Point system.^8^ All patients were treated with standard therapy in accordance with current guidelines.^7^ A reference set of imaging in a large cohort of healthy control subjects was taken from the UK Digital Heart Project (https://digital-heart.org/).^9^

### Study design

**Figure 1:**
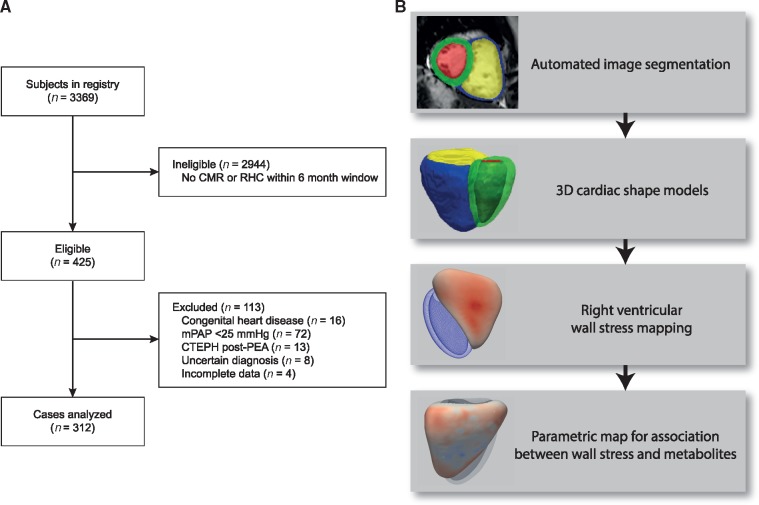
Study design. (*A*) Flowchart to show the patients included in the study. (*B*) Flowchart of the image analysis steps from automated CMR segmentation, shape modelling, and statistical parametric mapping. CMR, cardiac magnetic resonance imaging; CTEPH, chronic thromboembolic pulmonary hypertension; mPAP, mean pulmonary artery pressure; RHC, right heart catheterization; PEA, pulmonary endarterectomy.

A flowchart of the patients recruited to the study and the steps in image processing are shown in *Figure 1*. We used automated computational image analysis to create a biomechanical model of the heart from CMR images in patients with PH. We then modelled the relationship between haemodynamic parameters and 10 candidate metabolites, previously shown to be associated with outcome in PAH,^6^ on the structure and function of the right ventricle across all PH groups. Lastly, we assessed the relationship between RV wall stress and survival.

### Imaging

CMR imaging to assess biventricular function was performed according to a standard clinical protocol.^10^ Imaging was performed on a 1.5T Philips Achieva (Best, Netherlands) and stored on an open-source database.^11^ Conventional manual volumetric analysis was performed using commercial cardiac analysis software to a standard published protocol.^12^ Indexed RV end-diastolic and end-systolic volumes were calculated and indexed stroke volume and ejection fraction (RVEF) derived. Papillary muscles and trabeculae were included in the cavity volume. Indexed RV mass (RVMI) excluded the interventricular septum. Ventricular mass index (VMI), or Fulton’s index, was the ratio of RV mass to left ventricular mass index (including the interventricular septum).^13^ All mass and volume measurements were indexed to body surface area (BSA) estimated by the Mosteller formula.

### Three-dimensional image analysis

All image processing was performed in Matlab (Mathworks, Natick, MA, USA). We took the short-axis cines for each PH patient and automatically aligned them by minimizing the intensity differences between each slice. Prior knowledge for the supervised machine learning segmentation algorithm comprised manually-annotated images at end diastole and end-systole in 57 PH patients, complementing the UK Digital Heart Project atlases, to extend accurate analysis of both shape and motion to RV-overload phenotypes.^9^ Each voxel in the PH atlases was assigned five labels (LV/RV cavity or myocardium, and background) using freely available software (ITKsnap, National Library of Medicine’s Insight Segmentation and Registration Toolkit). A multi-atlas approach utilized the entire dataset of labelled atlases rather than relying on a model-based average representation.^14^ An approximate graph search was performed to find correspondences between small cubic regions, or patches, in the image to be segmented and the database of labelled atlases. Spectral embedding, using a multi-layered graph of the images, captured global shape properties of the heart. Finally, we estimated anatomical patch correspondences based on a joint spectral representation of the image and atlases.^15^^,^^16^ The final segmentations were co-registered to an average template surface mesh, where vertex density was determined by the curvature at each sampling point, allowing cardiac shape or function within the population to be compared in a common space.

This resulted in a statistical model describing within-population spatial variation in RV geometry at approximately 20 000 vertices. The right ventricular inlet, outlet and apical/trabecular regions were also demarcated on the template image. WT was calculated as the distance between the endocardial and epicardial surfaces perpendicular to a mid-wall plane. Relative wall thickness refers to WT corrected for variation in ventricular volume following a scale transformation of the right ventricle. Curvature tensors were derived using an algorithm that estimates the curvature of a smooth surface from a sampled polyhedral approximation.^17^ Excursion was defined as the distance between corresponding anatomical points at end-systole and end-diastole.^18^ Geometric variation in RV shape was quantified by vertex-wise comparison to a mean template derived from our reference population of healthy adults.

The segmentation algorithms and template mesh are freely available at https://github.com/UK-Digital-Heart-Project/PHsegment.

### Metabolomics

Venous blood samples were put on ice, centrifuged (1300*g*, 15 min) within 30 min and stored at −80°C. Metabolomic profiling by ultra-performance liquid chromatography mass spectrometry was conducted by Metabolon (Durham, NC, USA), which provided semi-quantitative assessment of 949 named and 467 unnamed metabolite levels, annotated with pathways.

For the present study, we considered 10 candidate metabolites that had previously been identified as both discriminating PAH patients from symptomatic controls independent of confounding factors and being predictive of survival.^6^ These metabolites were dehydroepiandrosterone sulfate (DHEA-S), N2,N2-dimethylguanosine (N2DMG), N1-methylinosine (N1MI), 3-hydroxy-3-methylglutarate (3H3MG), N-acetylmethionine (NAM), N-formylmethionine (NFM), fumarate (FMR), 1-linoleoyl-2-eicosapentaenoyl-GPC (18:2/20:5) (1L2EP), sphingomyelin (d18:1/20:0, d16:1/22: 0) (SM), and N-acetyltaurine (NAT). Data for creatinine and bilirubin were also recorded as these were found to be the most common confounders associated with metabolite levels.^6^

### Right ventricular wall stress

Wall stress is a measure of the effect of hydraulic load on the myocardium and depends on the interaction between central pressure and the instantaneous geometry of the heart. We used an approach for modelling wall stress based on earlier work on the left ventricle in dilated cardiomyopathy.^19^ We used a modified Laplace formula that, in contrast with the Young–Laplace law, does not assume that WT is negligible.^20^ Wall stress was derived from the invasive systolic pulmonary artery pressure (sPAP), regional inner radius of curvature (*R*), and the corresponding end-systolic WT at each vertex using the following equation:

$$\text{Wall stress}=0.133\;\times \text{ sPAP }\times \;\frac{R}{2\text{WT }\times \left(1+\frac{\text{WT}}{2R}\right)}$$

A conversion factor of 0.133 was used to express the results in kN/m^2^. Wall stress values were calculated for each vertex in the 3D model.

### Statistical analysis

Data were analysed in R version 3.4.0 using RStudio Server version 1.043 (Boston, MA, USA). Variables were expressed as percentages if categorical, mean ± standard deviation if continuous and normal, and median ± interquartile range (IQR) if continuous and non-normal. We used statistical parametric mapping to visualize the strength of association between metabolomics markers and patterns of regional wall stress. We did this by fitting univariate regression models at each vertex of the 3D cardiac mesh—a method which has been previously validated using both clinical and synthetic data.^21^ We adjusted for the following covariates: age, sex, ethnicity, BSA, PH group, and time from diagnosis. The metabolite relationships were further corrected for creatinine and bilirubin. We used a robust approach to inference, in which a consensus is drawn from multiple random data parcellations prior to permutation testing (Supplementary data online, *Figure S1*).^22^ Multiple testing correction was achieved by controlling the false discovery rate to 5% using the Benjamini–Hochberg^23^ procedure with models corrected jointly.^24^ RV regions where the association between variables was significant (*P *<* *0.05) were reported by the maximum *β* coefficient and the percentage area of RV free wall within the significance threshold.

The performance of RV wall stress as a predictor of survival was tested using a Cox proportional hazards multiple regression analysis (using the first principal component of the 3D wall stress data) with all-cause mortality as the outcome variable. Survival was defined as the time between date of the CMR imaging and death from any cause.

## Results

We report data from 312 PH subjects (47.1% female, mean age 60.8 ± 15.9 years), of which 182 (51.1% female, mean age 58.6 ± 16.8 years) had metabolomics (within a median 11 months of CMR), in whom we modelled the relationship between RV phenotype, haemodynamic state, and the pre-defined metabolites. Imaging in 1985 healthy volunteers (55.2% female, mean age 42.2 ± 13.2 years) was used as a normal reference for ventricular geometry. In all subject groups, segmentation was successful and no data were excluded from analysis. Baseline characteristics are given in *Table 1*.

**Table 1:** Summary of baseline patient characteristics

	All PH patients	PH patients with metabolomics	Healthy controls
*N*	312	182	1985
Age	60.8 ± 15.9	58.6 ± 16.8	42.2 ± 13.2
Female sex (%)	47.1	51.1	55.2
Body surface area (m^2^)	1.86 ± 0.25	1.9 ± 0.3	1.82 ± 0.21
Ethnicity, *n* (%)			
Caucasian	248 (79.5)	152 (83.5)	1379 (69.5)
South Asian	14 (4.5)	8 (4.4)	305 (15.4)
African Black	20 (6.4)	7 (3.8)	211 (10.6)
Others	30 (9.6)	15 (8.2)	90 (4.5)
PH group^a^, *n* (%)			
Pulmonary arterial hypertension	99 (31.7)	72 (39.6)	
PH due to left heart disease	42 (13.5)	7 (3.8)	
PH due to lung disease	12 (3.8)		
CTEPH	150 (48.1)	103 (56.6)	
PH with unclear mechanisms	9 (2.9)		
Functional			
Six-minute walk distance (m)	259.6 ± 157.8	276 ± 156.4	
WHO/NYHA functional class, I/II/III/IV	3/39/220/50	1/28/124/29	
Baseline haemodynamics			
Pulmonary capillary wedge pressure (mmHg)	13.8 ± 6.1	10.9 ± 7.2	
Mean pulmonary artery pressure (mmHg)	44.5 ± 13.2	46.7 ± 13.3	
Pulmonary vascular resistance (Woods units)	8.5 ± 5.5	9.3 ± 5.9	
Cardiac index (L/min/m^2^)	2.4 ± 0.9	2.3 ± 0.9	
Volumetry			
RV end-diastolic volume (mL)	194.1 ± 72.4	196.1 ± 69.1	164.0 ± 38.3
RV end-diastolic volume (indexed) (mL/m^2^)	105.2 ± 40	106.2 ± 39.4	89.3 ± 15.8
RV stroke volume (mL)	73.8 ± 28.3	69.8 ± 27.2	94.5 ± 20.2
RV stroke volume (indexed), (mL/m^2^)	40.1 ± 15.9	37.7 ± 15.4	51.6 ± 8.3
RV ejection fraction (%)	40.5 ± 13.6	37.8 ± 13.4	58.2 ± 5.9
RV mass (g)	49.2 ± 15.3		

Variables are shown for the entire UK group and those with metabolomics as well as the healthy controls.

Values are expressed as mean ± standard deviation.

CTEPH, chronic thromboembolic PH; NYHA, New York Heart Association; PH, pulmonary hypertension; RV, right ventricle.

a Dana Point classification.

### Three-dimensional phenotypes

**Figure 2:**
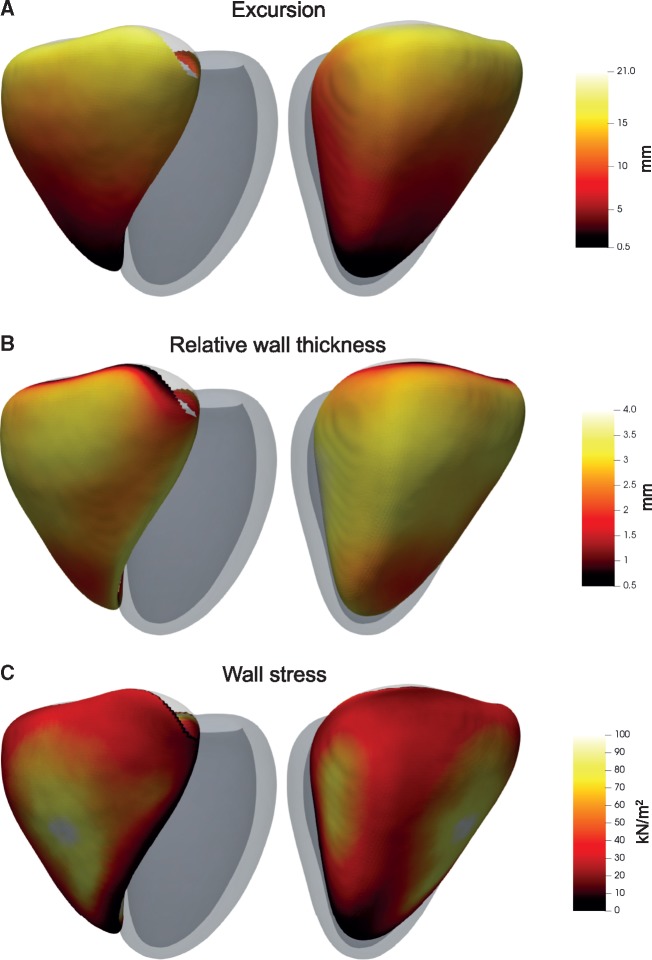
Three-dimensional models of the right ventricle in PH patients. Co-registered phenotypic data were used to create a map of RV features across the population. The RV is shown from two viewpoints with the left ventricle represented in grey. The median (*A*) systolic excursion, (*B*) relative wall thickness, and (*C*) wall stress at each corresponding anatomical point throughout the population (*n* = 312) is shown. These phenotypic data were then used in 3D regression models with haemodynamic variables or metabolomics levels as explanatory variables.

The 3D phenotypic characteristics of the right ventricle are shown in *Figure 2* and summary variables by anatomic region in Supplementary data online, *Table S1*. Median RV WT was 3.0 mm (IQR 2.6–3.4 mm). The RV free wall in the inlet region had the greatest absolute (3.2, IQR 2.9–3.6 mm) and relative WT (3.1, IQR 2.7–3.4 mm). Compared with healthy adults the RV outflow showed the greatest regional expansion in shape (1.6, IQR −0.8 to 4.0 mm) with increased excursion (13.9, IQR 10.6–17.5 mm) and higher wall stress (43.5, IQR 28.0–66.7 kN/m^2^). These comparisons, between apical/trabecular, inlet and outlet, were all significant (*P *<* *2.2 × 10^−16^). Overall, median RV wall stress was 39.0 kN/m^2^ (IQR 24.1–62.3 kN/m^2^).

**Figure 3:**
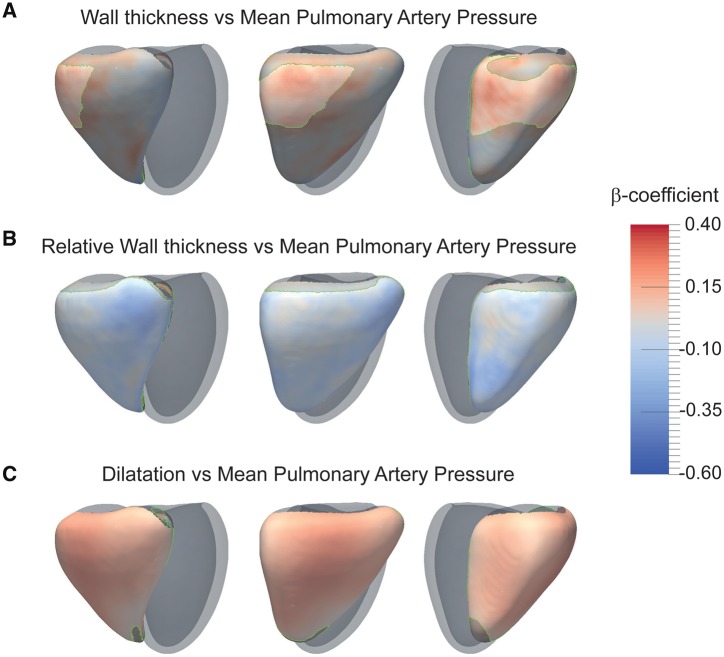
Statistical models of the right ventricle in PH patients. The strength of relationship between the stated variables is shown by 3D maps of standardized *β* coefficients with a significance threshold outlined in green. The RV is shown from three viewpoints with the left ventricle represented in grey. Relationships between mPAP and (*A*) wall thickness, (*B*) relative wall thickness, and (*C*) volume (*n* = 312). A positive regional relationship with mPAP is indicated in red and a negative relationship in blue. mPAP, mean pulmonary artery pressure.

Maps showing the relationship between phenotypic variation and mPAP are shown in *Figure 3*. RVMI was positively associated with mPAP (*β* = 0.13, *P = *0.03), and the strongest relationship between mPAP and WT was at the RV inlet (*β* = 0.29, significant area = 28% of RV free wall). RVMI/RVEDVI was negatively associated with mPAP (*β* = −0.18, *P = *0.0007) with a corresponding negative association between mPAP and relative WT throughout the free wall (*β* = −0.38, area = 88%). VMI, an indicator of remodelling,^25^ was positively associated with mPAP (*β* = 0.41, *P *=* *8.9 × 10^−15^). Patients’ mPAP was positively associated with right ventricular end-diastolic volume (RVEDV) (*β* = 0.23, *P *=* *8.5 × 10^−5^), corresponding to a global expansion in RV shape (*β* = 0.29, area = 95%). Regional systolic excursion was negatively associated with mPAP (*β* = −0.33, area = 73%). Wall stress and RVEF were inversely associated (*β* = −0.63, area = 97%) (Supplementary data online, *Figure S2*). RVEF itself was negatively associated with mPAP (*β* = −0.5, *P *<* *2 × 10^−16^).

### Metabolites associated with global variables and wall stress

**Figure 4:**
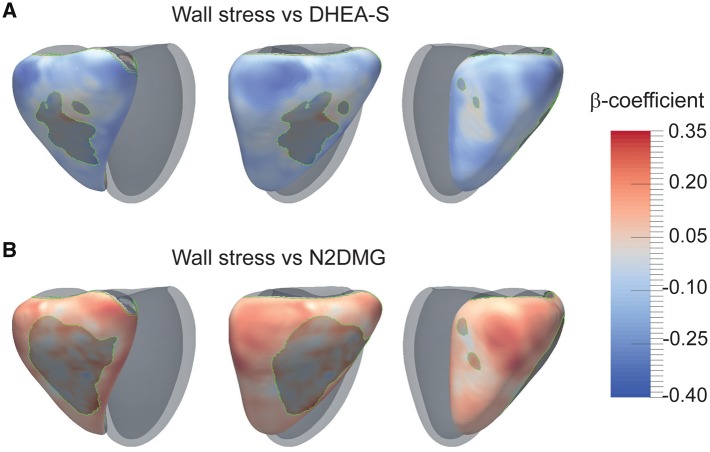
Statistical models of the relationship between right ventricular wall stress and two metabolites in PH patients. The strength of relationship between metabolite concentration and RV end-systolic wall stress is shown by 3D maps of standardized *β* coefficients with a significance threshold outlined in green (*n* = 182). The RV is shown from three viewpoints with the left ventricle represented in grey. A positive relationship between metabolite level and regional wall stress and is indicated in red and a negative relationship in blue. DHEA-S, dehydroepiandrosterone sulfate; N2DMG, N2,N2-dimethylguanosine.

The relationship between metabolite concentration and cardiac variables is shown in *Table 2*. Maps of the relationship between wall stress and metabolites are shown in *Figure 4*, and Supplementary data online, *Figures S3* and *S4*. Of the 10 metabolites evaluated all except 1L2EP were significantly related to RVEF (*P *<* *0.05), each with a negative association (*β* = −0.21 to −0.28) apart from DHEA-S which had a positive relationship (*β* = 0.31). Five of these metabolites were also positively associated with wall stress (*β* = 0.28–0.34), again with DHEA-S showing a negative association (*β* = −0.40), with at least 70% of the RV free wall reaching significance (*P *<* *0.05). Metabolites were associated with a reduced RVM: RVEDV ratio indicating eccentric remodelling.

**Table 2:** Metabolite associations

Metabolite	Pathway	RVM	RVEDV	RVM/RVEDV	RVEF	VMI	3D wall stress
DHEA-S	Steroid	–	−0.26**	0.18*	0.31***	−0.21**	−0.4, 86.8%*
N2DMG	Purine, Guanine	–	0.18*	−0.17*	−0.28***	–	0.34, 73.1%*
N1MI	Purine, Hypo-Xanthine/Inosine	–	–	–	−0.21**	–	0.28, 87.2%*
3H3MG	Mevalonate	–	–	–	−0.25**	0.18*	0.32, 81.7%*
NAM	Met, Cys, SAM & Tau	–	–	−0.18*	−0.22**	–	0.31, 81.9%*
NFM	Met, Cys, SAM & Tau	–	0.17*	−0.14*	−0.23**	−0.17*	0.32, 69.7%*
Fumarate	TCA cycle	–	0.26**	−0.24**	−0.26**	0.22*	–
1L2EP	Phospholipid	–	–	–	–	–	–
SM	Sphingolipid	–	–	–	0.2*	−0.24**	–
NAT	Met, Cys, SAM & Tau	–	0.23*	−0.17*	−0.18*	–	–

Adjusted *P*-value:

* <0.05;

** <0.01;

*** <0.001.

The relationship between metabolite concentration and phenotypic variables corrected for false discovery rate (presented as *β* coefficient with adjusted *P-*value, including area% for wall stress mapping).

3D, three-dimensional; DHEA-S, dehydroepiandrosterone sulfate; 3H3MG, 3-hydroxy-3-methylglutarate; 1L2EP, 1-linoleoyl-2-eicosapentaenoyl-GPC (18:2/20:5); NAM, N-acetylmethionine; NAT, N-acetyltaurine; NFM, N-formylmethionine; N1MI, N1-methylinosine; N2DMG, N2, N2-dimethylguanosine; RVEDV, right ventricular end-diastolic volume; RVEF, right ventricular ejection fraction; RVM, right ventricular mass; RVM/RVEDV, RVM corrected for RVEDV; VMI, ventricular mass index; SM, sphingomyelin (d18:1/20:0, d16:1/22:0); –, not significant.

### Survival

In multiple regression analysis, RV wall stress predicted survival independently of age, sex, BSA, time from diagnosis, and PH group (hazard ratio = 1.27, 95% confidence intervals 1.01–1.61, *P *=* *0.04).

## Discussion

We developed a fully automated approach for 3D modelling of the right ventricle in patients with PH enabling a comprehensive quantitative analysis of the complex relationship between elevated afterload, serum biomarkers and structural adaptation. We found that biomechanical overload, indicated by rising RV wall stress, is associated with altered levels of circulating biomarkers that report on energy metabolism and stress-response pathways. These results identify putative pathways that may influence RV adaptive mechanisms to elevated mechanical stress.

Integrative mathematical and statistical models of cardiac anatomy and physiology can play a vital role in understanding cardiac disease phenotypes and planning therapeutic strategies,^26^ but previous work in this field has largely been confined to modelling the left ventricle. Here, we develop a powerful automated approach for modelling the geometry and function of the right ventricle in PH patients—enabling inferences about the relationship between biomarkers and complex cardiovascular phenotypes to be made within large populations. Invasive measurements in PH indicate that wall stress is the primary determinant of tissue oxygen consumption under adverse biomechanical conditions and the main driver of neurohumoral activation in myocytes.^27^^,^^28^ We report the first results of 3D population mapping of wall stress in PH demonstrating strong regional variations across the freewall acting as a stimulus for adaptive myocardial hypertrophy and eccentric remodelling. We also observed that RVEF and wall stress were closely and inversely related to each other—reflecting the dependence of ejection fraction on wall stress rather than as an indicator of RV systolic function.^29^

The strongest associations between metabolite levels and regional wall stress were identified for decreased levels of the sulfated steroid DHEA-S and increased levels of circulating modified nucleosides (N2, N2-dimethylguanosine, N1-methylinosine). Cardiovascular disease patients with lower DHEA-S levels are reported to have a poorer prognosis although the mechanisms for mediating health outcomes remain unclear,^30^ DHEA-S may act as a functional antagonist of glucocorticoids and abnormalities in the regulation and metabolism of insulin, sex hormones, adipokines, and lipids have been found in both animal and human studies of PH.^31^ Ours is the first study to report that DHEA-S is also associated with phenotypic evidence of prognostically-important cardiac maladaptation in PH. DHEA prevents and reverses chronic hypoxic PH in rat models and its therapeutic potential in humans is under investigation.^32^^,^^33^

Transfer RNA abundance is dynamically regulated and these molecules are involved in adaptive translational pathways and stress signalling in diverse disease states.^34^ Our findings indicate that tRNA biology is closely linked to progressive states of RV decompensation across PH disease groups. Significant associations between adverse wall stress patterns and pathways related to cellular energy production were also observed in our population. In PH, the myocardium shifts from oxidative phosphorylation to anaerobic glycolysis.^35^ Such metabolic remodelling has been associated with a transition from a compensated to decompensated state in PH,^36^ and our findings show that dysfunctional energy metabolism is related to biomechanical overload. Restoration of glucose oxidation by dichloroacetate therapy has recently shown promise as a metabolic approach for reducing mPAP and improving functional capacity.^37^ This is one of a growing number of novel strategies to combat the deleterious effects of walls stress in PH that include improving oxygen delivery, restoring mitochondrial function, and modifying neurohormonal modulation.^2^

### Limitations

This study has strengths and limitations. Our computational analysis provides a powerful framework for understanding the relationship between RV physiology and putative biomarkers across large PH populations. Our algorithms readily scale to large populations and in future may enable high-throughput screening of disease-modifying molecular pathways across health and disease, as well as a direct use in providing quantitative visualizations of the right ventricle in clinical practice. The models could be further refined with knowledge of fibre orientations and myocardial mechanical properties. The metabolites investigated had been previously shown to discriminate healthy and disease control subjects and independently predict survival—and we found convincing associations between these biomarkers and RV overload in a broader population of PH patients. However, we cannot establish causal interrelationships between these biomarkers and right heart dysfunction, and nor did we have direct tissue or transvenous pulmonary sampling to determine the source or tissue concentrations of these metabolites. Our cohort also represented a relatively diverse cohort receiving a range of standard therapies. Lastly, a significant number of potentially eligible patients did not have CMR near the time of RHC reflecting current clinical practice.

## Conclusion

In conclusion, wall stress influences outcomes in PH and is associated with metabolic pathways that report on energy metabolism and cellular stress response. Several of these pathways are tractable as therapeutic targets for reducing wall stress and warrant further investigation.

## Supplementary Material

jey175_Supplementary_Data
