# Supplementary material for: Metabolic pathways associated with right ventricular adaptation to pulmonary hypertension: 3D analysis of cardiac magnetic resonance imaging
Source: Eur Heart J Cardiovasc Imaging. 2018 Dec 7;20(6):668–76. doi: 10.1093/ehjci/jey175 (PMC6529902; doi:10.1093/ehjci/jey175)
Supplement: jey175_Supplementary_Data [file jey175_supplementary_data.docx]

**Metabolic pathways associated with right ventricular adaptation to pulmonary hypertension: three dimensional analysis of cardiac magnetic resonance imaging**

SUPPLEMENTARY MATERIAL

|  | **Median** | **Interquartile range** | **Lower quartile** | **Upper quartile** |
| --- | --- | --- | --- | --- |
| **End-diastolic wall thickness (mm)** | | | | |
| Inlet | 3.24 | 0.76 | 2.87 | 3.63 |
| Outlet | 2.97 | 0.75 | 2.59 | 3.34 |
| Apical | 2.88 | 0.80 | 2.48 | 3.28 |
| Overall | 3.03 | 0.82 | 2.62 | 3.43 |
| **Relative wall thickness (mm)** | | | | |
| Inlet | 3.07 | 0.71 | 2.70 | 3.41 |
| Outlet | 2.79 | 0.74 | 2.39 | 3.13 |
| Apical | 2.72 | 0.69 | 2.37 | 3.06 |
| Overall | 2.85 | 0.76 | 2.45 | 3.21 |
| **Dilation (mm)** | | | | |
| Inlet | 1.13 | 5.83 | -1.64 | 4.19 |
| Outlet | 1.64 | 4.77 | -0.82 | 3.95 |
| Apical | 1.19 | 4.80 | -1.16 | 3.64 |
| Overall | 1.95 | 5.35 | -0.74 | 4.61 |
| **Excursion (mm)** | | | | |
| Inlet | 11.80 | 7.22 | 8.52 | 15.74 |
| Outlet | 13.85 | 6.85 | 10.62 | 17.47 |
| Apical | 7.00 | 5.67 | 4.41 | 10.08 |
| Overall | 10.29 | 8.50 | 6.42 | 14.92 |

| **Curvature (mm^-1^)** | | | | |
| --- | --- | --- | --- | --- |
| Inlet | 0.04 | 0.03 | 0.03 | 0.05 |
| Outlet | 0.03 | 0.02 | 0.02 | 0.04 |
| Apical | 0.03 | 0.04 | 0.02 | 0.06 |
| Overall | 0.03 | 0.03 | 0.02 | 0.05 |

| **Wall stress (kN/m^2^)** | | | | |
| --- | --- | --- | --- | --- |
| Inlet | 35.54 | 31.96 | 22.72 | 54.68 |
| Outlet | 43.49 | 38.69 | 28.03 | 66.72 |
| Apical | 38.30 | 43.03 | 22.30 | 65.33 |
| Overall | 38.95 | 38.08 | 24.17 | 62.25 |

**Table S1. Characteristics of three dimensional phenotypes.** Median, interquartile range and quartiles for each of the three dimensional imaging phenotypes.


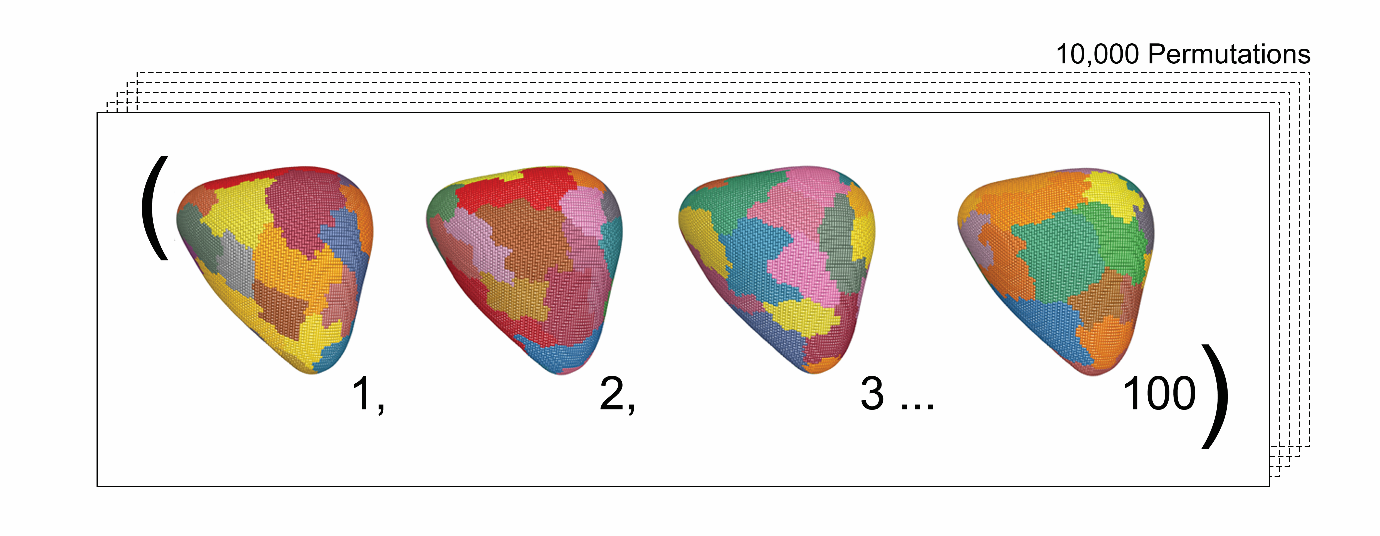


**Figure S1. Randomized parcellation based inference on cardiac data.** A variant of Ward’s hierarchical agglomerative clustering was used on features, finding similarities but with added spatial and contiguity constraints. Cohesion between clusters was based on minimising Euclidean distances in between clusters. A predefined clustering cut-off was applied to this unsupervised dimensionality reduction tool. This was then implemented on bootstrapped samples of the cohort to generate 100 randomized parcellations each with 50 parcels. The mean signal was extracted for each parcel, parcellation and subject to generate an n × 50 × 100 array of mean signal. The statistical design was tested on each parcel generating a t-statistic map and this was repeated 10,000 times with permutations to map the distribution under the null hypothesis. A family-wise error rate p-value map was produced which was then further corrected for multiple testing using Benjamini-Hochberg procedure to a false discovery rate of 5%.


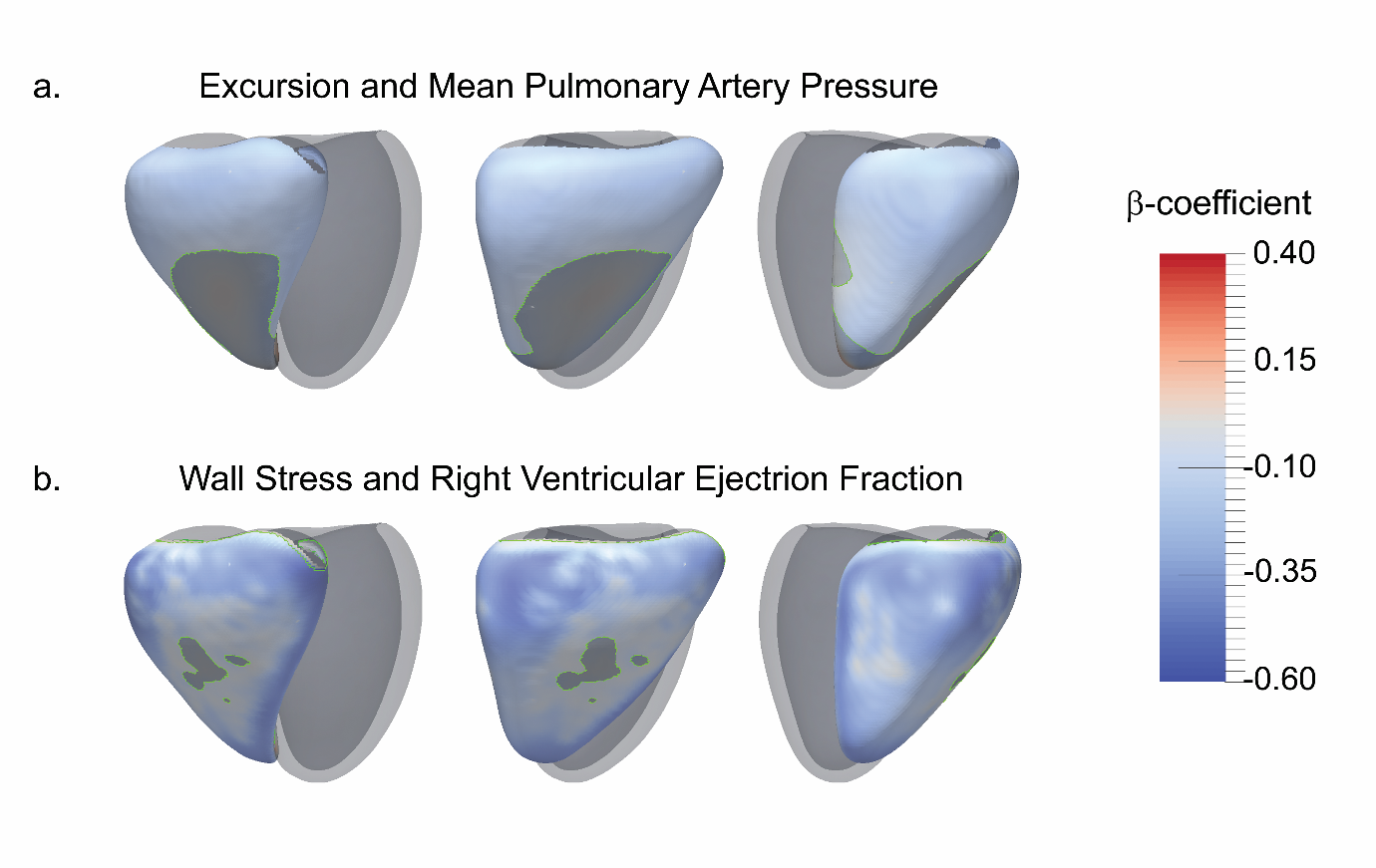


**Figure S2. Statistical parametric models of the right ventricle in pulmonary hypertension.** Three-dimensional maps of standardized β coefficients with a significance threshold outlined in green. The right ventricle is shown from three viewpoints with the left ventricle represented in grey. Relationships between (a) mean pulmonary artery pressure and excursion, and (b) right ventricular ejection fraction and wall stress, are shown (n=182). The extent of the negative relationship is represented by the blue colour spectrum according to the β-coefficient legend.


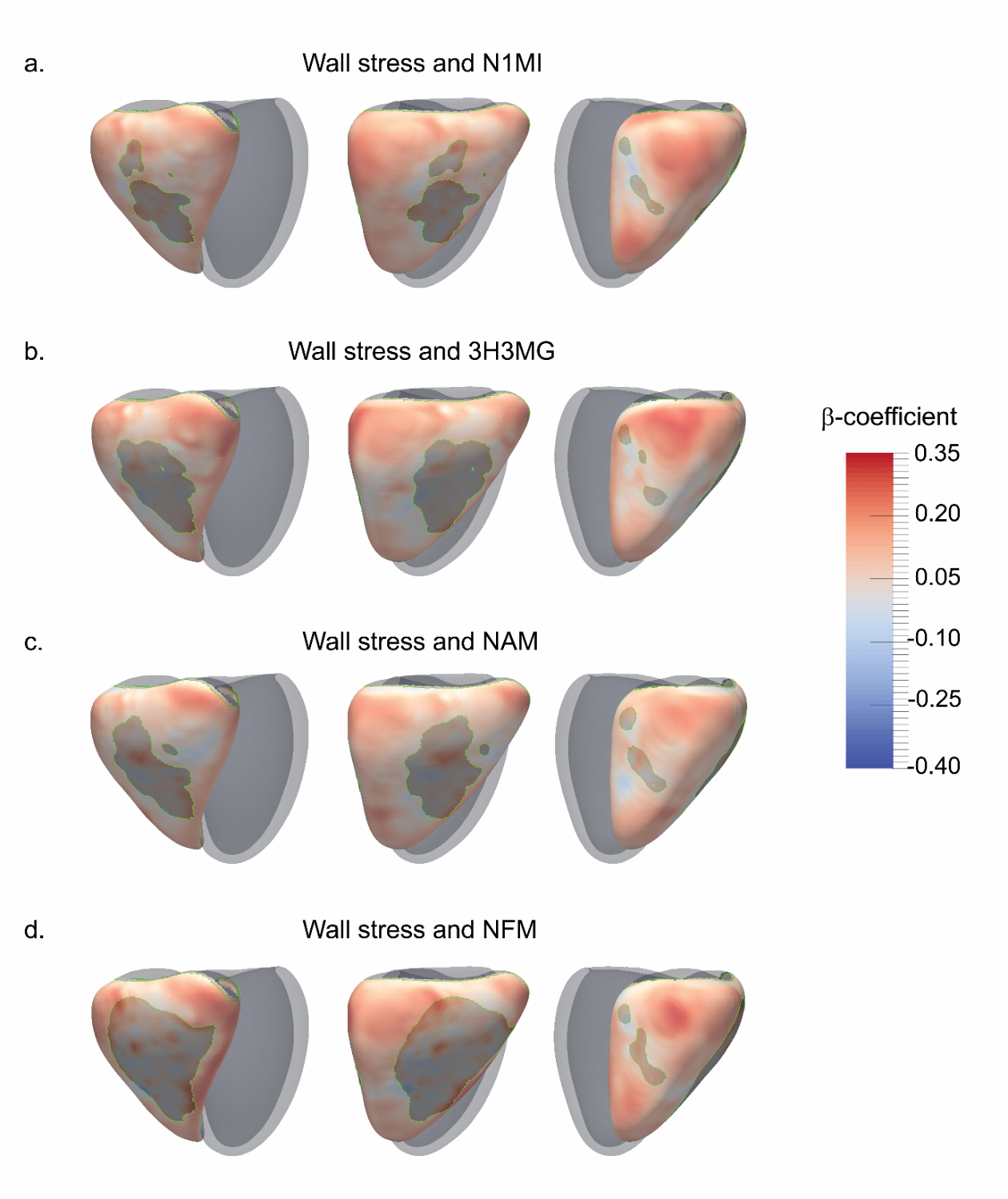


**Figure S3. Metabolites with significant relationships with wall stress.** The strength of the relationship between metabolite concentration and right ventricular end-systolic wall stress is shown by three-dimensional maps of standardized β coefficients with a significance threshold outlined in green. The right ventricle is shown from three viewpoints with the left ventricle represented in grey (n=182). (Please also see Figure 4 in the main paper). A positive relationship between metabolite level and regional wall stress and is indicated in red and a negative relationship in blue. N1MI, N1-methylinosine; 3H3MG, 3-hydroxy-3-methylglutarate, NAM, N-acetylmethionine; NFM, N-formylmethionine.


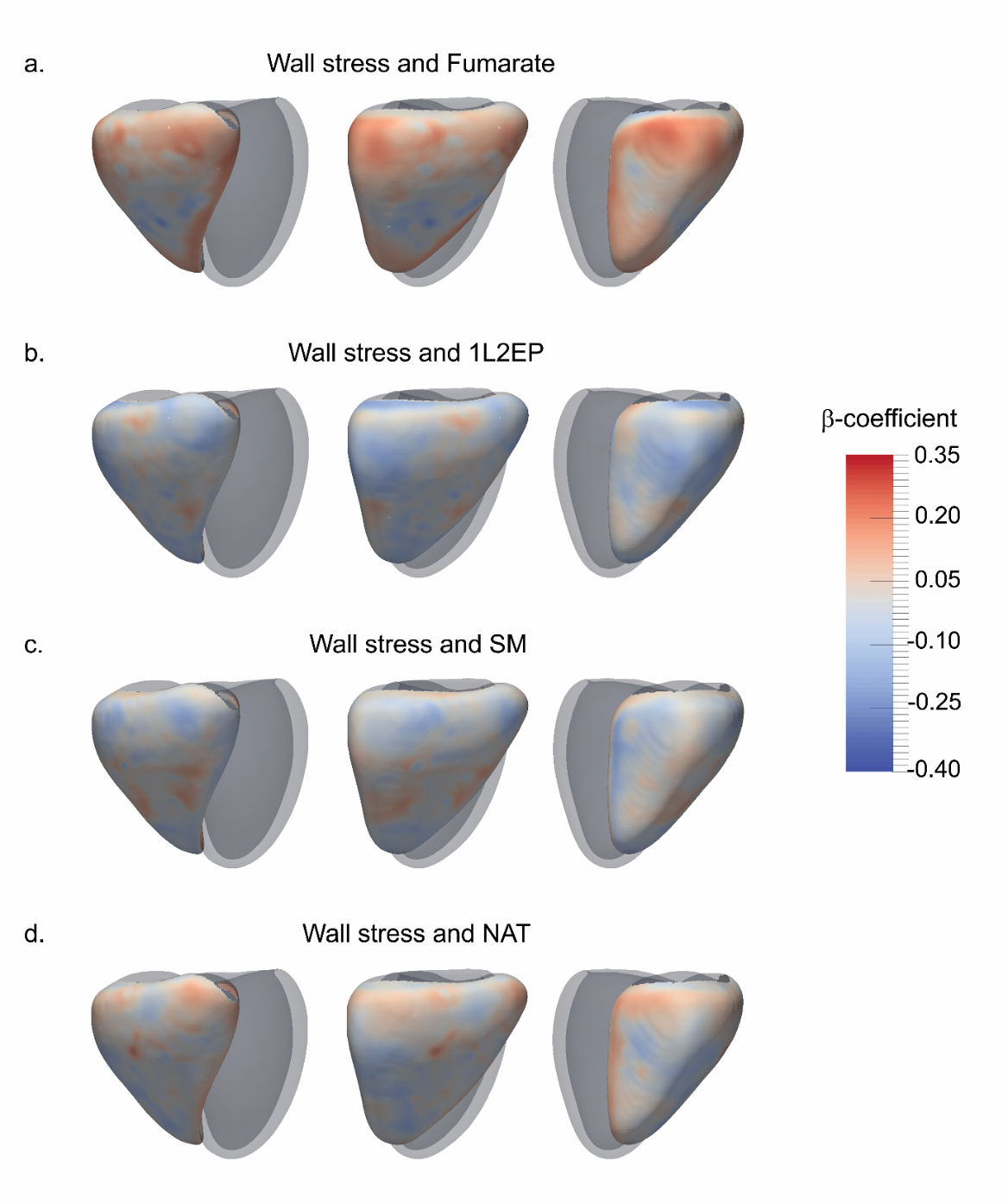


**Figure S4. Metabolites with no significant relationships with wall stress.** Models demonstrating the strength of relationship between metabolite concentration and right ventricular end-systolic wall stress, but not reaching a significance threshold, are shown by three-dimensional maps of standardized β coefficients (n=182). The right ventricle is shown from three viewpoints with the left ventricle represented in grey. 1L2EP, 1-linoleoyl-2-eicosapentaenoyl-GPC (18:2/20:5)*; SM, sphingomyelin (d18:1/20:0, d16:1/22:0)*; NAT, N-acetyltaurine; *, metabolites identified by mass and fragmentation analysis but yet to be confirmed with reference standards...
